# Clinical diagnostic reference levels in neuroradiology based on clinical indication

**DOI:** 10.1093/rpd/ncae113

**Published:** 2024-05-03

**Authors:** Antar Aly, Virginia Tsapaki, Ayman Zakaria Ahmed, Ahmed Own, Satya Patro, Huda Al Naemi, Mohammad Hassan Kharita

**Affiliations:** Medical Physics Section, Hamad Medical Corporation, Doha 3050, Qatar; Radiology Department, Weill Cornell Medicine, Doha 24144, Qatar; Medical Physics Department, Konstantopoulio Hospital, 142 33 Nea Ionia, Athens, Greece; Neurosurgery Department, Hamad Medical Corporation, Doha 3050, Qatar; Neurosurgery Department, Hamad Medical Corporation, Doha 3050, Qatar; Neurosurgery Department, Hamad Medical Corporation, Doha 3050, Qatar; Radiology Department, Weill Cornell Medicine, Doha 24144, Qatar; Medical Physics Section, Hamad Medical Corporation, Doha 3050, Qatar

## Abstract

This study focuses on patient radiation exposure in interventional neuroradiology (INR) procedures, a field that has advanced significantly since its inception in the 1980s. INR employs minimally invasive techniques to treat complex cerebrovascular diseases in the head, neck, and spine. The study establishes diagnostic reference levels (DRLs) for three clinical indications (CIs): stroke (S), brain aneurysms (ANs), and brain arteriovenous malformation (AVM). Data from 209 adult patients were analyzed, and DRLs were determined in terms of various dosimetric and technical quantities. For stroke, the established DRLs median values were found to be 78 Gy cm^2^, 378 mGy, 118 mGy, 12 min, 442 images, and 15 runs. Similarly, DRLs for brain AN are 85 Gy cm^2^, 611 mGy, 95.5 mGy, 19.5, 717 images, and 26 runs. For brain AVM, the DRL’s are 180 Gy cm^2^, 1144 mGy, 537 mGy, 36 min, 1375 images, and 31 runs. Notably, this study is unique in reporting DRLs for specific CIs within INR procedures, providing valuable insights for optimizing patient safety and radiation exposure management.

## Introduction

Many medical conditions that could not be treated effectively 15 y ago can now be treated curatively using current endovascular techniques. Indeed, even within the field of interventional neuroradiology (INR), new technology and devices introduced within the past 5 y have allowed interventional neuroradiologists to increase the number of life-threatening cerebrovascular diseases that can be treated effectively [1]. With the immense and rapid evolution of medical imaging technology, the variety of techniques offered for interventional radiology (IR) has increased exponentially over the last few years, boosting the utilization of IR in the everyday clinical routine. Due to the use of X-ray imaging devices to execute IR procedures such as sophisticated angiography machines, there is a risk for stochastic or tissue reactions [2–5]. According to Sanchez *et al.* [6], the dose delivered to the brain of patients undergoing specific INR techniques may be relevant enough to produce radiation side effects and must be minimized as much as possible. A recent study suggests that the radiation doses associated with neuroradiological interventions can lead to deterministic effects on the skin in ~6% of cases and recommends systematic monitoring of doses [7].

One of the cornerstones of radiation protection in medical exposure and radiation dose optimization is the establishment of diagnostic reference levels (DRLs). This is specifically stated in the International Basic Safety Standards (BSS) [8], in the European BSS [9], and in many other publications [10–18]. DRLs have no direct linkage to the numerical values of the Commission’s dose limits or dose constraints. Ideally, they should be the result of a generic optimization of protection. In practice, this is unrealistically difficult, and it is simpler to choose the initial values as a percentile point on the observed distribution of doses to patients. The values should be selected by professional medical bodies (in conjunction with national health and radiological protection authorities) and reviewed at intervals that represent a compromise between the necessary stability and the long-term changes in the observed dose distributions. The selected values could be specific to a country or region. [11]. DRLs are considered mandatory in the European Union [9, 13], whereas the establishment of national DRLs is also mentioned in the IAEA BSS [8].

DRLs in most of the international literatures are set for certain anatomical regions [14]. However, the limitation of anatomical DRLs is that for one anatomical region of the patient’s body, more than one clinical indication (CI) is applicable. Each of these CIs usually requires a different protocol or technique to answer the clinical question, resulting in varying patient exposure. Recently, the term clinical DRL was introduced. A European Study on Clinical DRLs (EUCLID European project) was also recently funded by the European Commission with the aim to define clinical DRLs for the most important CIs from the radiation protection perspective across Europe [15]. Defining DRLs specifically for IR procedures, even for anatomical regions and not CIs, is indeed challenging. There are no established national diagnosticreference levels (DRL). Another poor practice reflects the lack of patient dose records inradiological practice per year and population. [15]. There are several reasons that hinder the establishment of DRL in IR, such as clinical problem complexity, operator experience and skills, access to the target lesion, type of catheters used, X-ray equipment technical characteristics, and radiation protection tools, such as lead aprons, eye googles, zero gravity apron, and ceiling-suspended lead screen shielding.

## Objectives

1) To evaluate patient radiation doses in INR procedures based on CI.

2) To establish clinical DRLs for the CIs of stroke (S), brain aneurysm (AN), and brain arteriovenous malformation (AVM) in HMC, State of Qatar.

## Materials and Methods

### Study design

In an attempt to evaluate patient radiation exposure based on CI and investigate the possibility of establishing institutional clinical DRLs in the State of Qatar, the first meeting was held in February 2019 with representatives of the respective radiology departments (the main health care provided in Qatar), Hamad Medical Corporation (HMC) Medical Physics Section, and the Medical Physics Society. During the initial meeting, the CIs to focus on were decided and chosen because of the high frequency in the state of Qatar and because of the possible increased radiation exposure of patients [17].

Due to the complexity of the procedures, not only the dosimetric data (KAP, CDf, and CDl) but also all technical data (T, Ni, and Nr) will be part of DRLs. This is also commented in the latest ICRP Report 135, which provides guidelines for the establishment of DRLs [12]. According to the report, the use of multiple DRL quantities may help to identify the cause of possible overexposure, leading to simplifying the investigation thereafter. ICRP 135 reports recommend that all available data suitable for DRL quantities should be tracked [14].

The selected hospital is currently the only governmental healthcare provider that performs INR in Qatar; however, the total number of patients who underwent IR procedures is 209.

### Clinical indications included in the study

Three CI’s were identified to be included in the study: stroke (S), brain ANs, and brain AVM. These were all therapeutic (endovascular treatment and embolization) and were the following: (i) thrombectomy for treatment of S (mechanical or aspiration or both), (ii) endovascular treatment of AN by inserting various devices, such as coils, stents, balloons, or flow diversion devices, and (iii) endovascular treatment of AVM (embolization by liquid embolic material, such as Qnyx or Squid).

Occlusion of a major cerebral blood vessel is an important cause of cerebral stroke. This should be managed as soon as possible to restore blood flow to the brain and prevent brain cell death. This can be done by giving thrombolytic agents that help to dissolve the thrombus (effective in 10%–30% of cases) or better by extracting the thrombus out by a special catheter and a stent retriever (mechanical thrombectomy) or aspirating the thrombus out with a large bore catheter (aspiration thrombectomy). Both endovascular techniques can be combined to re-establish the blood flow in the occluded vessel (effective in 85%–90% of cases) [18].

Treatment is done in an emergency in ruptured cases by the exclusion of the AN from circulation. The endovascular route is a minimally invasive method of treatment compared to conventional surgery, and it is done by simple coiling, stent-assisted coiling, balloon-assisted coiling, flow diverters, or parent artery occlusion. These techniques are the standard preferred treatment modalities all over the world, with excellent outcomes, low morbidity, and mortality associated with a shorter patient hospital stay [19].

Cerebral AVM is a common finding. Studies have proven that in every four persons, one will have a cerebral AVM with a 1.5% risk of bleeding throughout life. If the malformation bleeds, it requires treatment by surgical excision, radiation therapy, or better by, endovascular embolization. This method uses very small microcatheters guided by microwires to reach the malformation and fill it with liquid embolic material to occlude the malformation and its draining vein and abolish the risk of a second bleed, which could have bad clinical consequences. The success of embolization in complete occlusion of ruptured cerebral AVM reaches 60% with lower morbidity and mortality than other treatment modalities [20].

### Patient data collection

Retrospective data collection was done on 209 patients utilizing radiation dose monitoring (RDM) software. To ease uniform data collection and aid in data analysis, forms were produced and distributed in Excel format during the meeting. The patient’s personal data (gender, age, weight, height, etc.), technical parameters for the X-ray modality (fluoroscopy time (T), number of images (N), number of runs (r), and operator data (contact information, years of experience), were all intended to be included on the forms. The study only involved adult patients (16 y or older). The study’s emphasis was solely on adult patients because the workload for pediatric patients was insufficient.

The dosimetric quantities for the establishment were provided by the X-ray modality, and these were: kerma-area product (KAP) and cumulative air kerma dose (CD) at the reference point. The study received ethical approval from the corporation’s Medical Research Center. For the accurate establishment of clinical DRLs, the methodology described in International Commission on Radiological Protection (ICRP) Report 135 was strictly followed [12]. Only INR procedures with complete patient information were included. Examinations with missing or incoherent information were excluded to increase the power of statistical analysis. Quality control tests for the selected machines were done to ensure that the displayed dose descriptors were accurate before using them in this study. Quality control tests include image quality, entrance surface air-kerma rates (for selected standard imaging modes), kVp accuracy, beam quality, KAP meter and reference air-kerma accuracy, etc., all based on HMC quality control procedures for diagnostic X-ray equipment [13]. Finally, a subjective image quality evaluation was made for each patient included in the survey to ensure that image quality was adequate to answer the corresponding clinical problem. The INR procedures were performed by three neuroradiologists with 4–13 y of experience. All images were assessed for diagnosticity by experienced operators.

### X-ray equipment used

**Figure 1:**
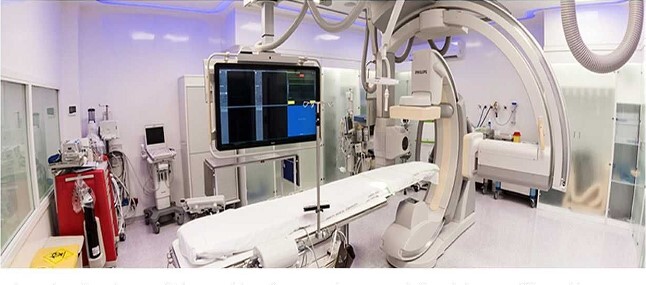
The angiography room with the state-of-the-art floor-mounted X-ray system (Allura Clarity FD20, Philips Healthcare, Best, Netherlands) installed in 2016 is shown.

All INR procedures in HMC hospital’s neurosurgical department are equipped with state-of-the-art technology biplane floor mounted (Allura Clarity FD20, Philips Healthcare, Best, Netherlands), which was installed in 2016 (Fig. 1). The system was equipped with a digital control console, incorporating preset anatomic/examination protocols, and an automatic exposure control (AEC) system. Exposure parameters are adjusted to achieve the appropriate exposure level to the image receptor, which, combined with postprocessing algorithms, offers optimum image quality for the diagnostic task for which the protocol is designated. The X-ray system displays radiation exposure in terms of KAP and CD. The hospital has had a well-established quality assurance program for many years and is accredited by the Joint Commission International (JCI). The accuracy of KAP indication was verified following established procedures within the quality assurance program of the hospital based on international norms [20]. The X-ray equipment has a state-of-the-art technology system feature for performing INR procedures, including dose reduction tools, such as last image hold, changing pulse rate, and frame rate, etc.

After data collection, a second meeting was held during which data were discussed, cleaned, typos were corrected, and clarifications were provided in order to proceed with data analysis. Once data cleaning was finalized, analysis was performed.

### Statistical analysis

Data were analyzed by the medical physicists to determine 75 percentile, mean, SD, min, max, and median of patient dose-related parameters. For each CI, the median dose was defined as the 50th percentile in dose distribution.

## Results

The data were collected retrospectively from January 2019 to March 2020. The study includes data from 209 adult patients undergoing endovascular treatment: 99 patients for stroke (S), 63 patients for brain AN, and 47 patients for AVM.

The mean age ± SD for the patient was estimated to be 52.8 ± 14, 43.8 ± 13, and 34 ± 10.8 for stroke, AN, and AVM, respectively. The majority of the patients were male; however, 24.9% were female and 75.1% were male patients for all the INR procedures.

As already mentioned elsewhere [17], 96% of the population is urban, with a median age of 32.3 y, and consists of expats that come to the country for work. Due to the huge influx of male workforce, women account for just 25% of the population. Thus, the population is distinctive with a considerable percentage of noticeably young male inhabitants coming from other countries of the world. This explains the reason why the three CIs were mostly male population.

Subjective image quality evaluation showed that in all patient cases, it was sufficient to answer the clinical question and perform the procedure. As mentioned in the Methods Section, the focus of the study was to establish DRLs based on CI considering that image quality is sufficient to provide the diagnostic information required, also recommended in the ICRP guidance report 135 [12]. The evaluation was performed immediately after the procedure by each neuroradiologist who executed the INR technique.

**Figure 2:**
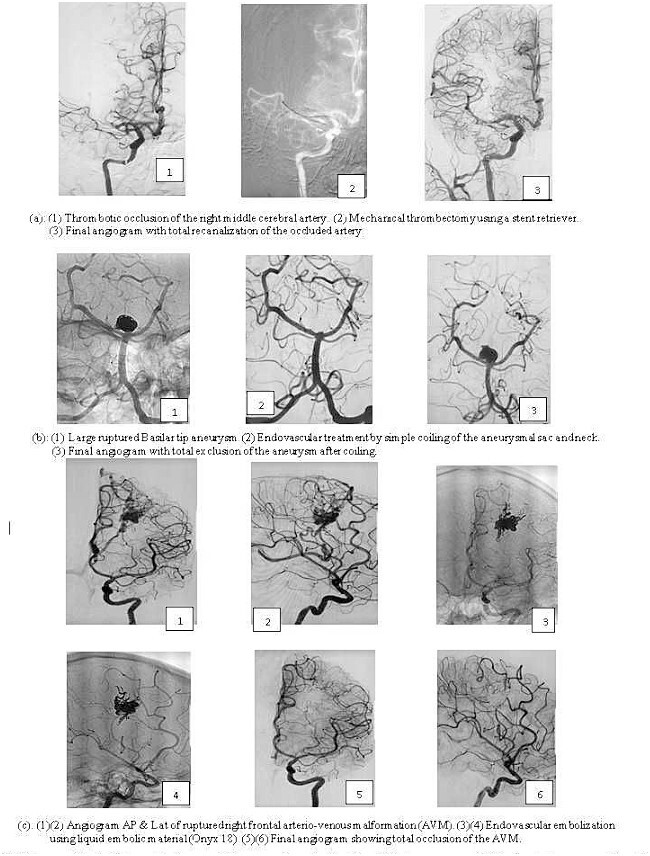
Images of typical interventional neuroradiology procedures for (a) stroke, (b) brain aneurysm, and (c) brain arteriovenous malformation.

Figure 2 shows the typical images of endovascular for (a) S, (b) AN, and (c) AVM, which include a final angiogram with total recanalization of the occluded artery, a final angiogram with total exclusion of the AN after coiling, and a final angiogram showing total occlusion of the AVM.

Table 1 shows patient dosimetric and other technical data for S, AN, and AVM, respectively. As the data did not follow a normal distribution, the 75 percentiles, median, and range of dosimetric data were estimated and shown here. Table 1 shows that treatment for stroke imparts the lowest dose to the patient both in terms of KAP (sum of KAP of frontal and lateral C-arm units) and in CD (frontal and lateral) and has the lowest fluoroscopy time, number of images, and runs. AVM imparts more than double KAP than stroke (78 mGy cm^2^ and 180 Gy cm^2^), more than triple CD (378 mGy and 1144 mGy), and fluoroscopy time (12 and 36 min). CD is the air kerma value at a specific point. Table 1 shows that CD (the summation of the frontal and lateral fluoroscopy) does not include the tissue backscatter. Data shows that AVM have increased CD value compared to AN and stroke, reaching levels of 1872 mGy under 2000 mGy, which is the threshold that may cause skin complications [21]. It is evident that even though all procedures involve therapeutical endovascular techniques, the CI affects largely the radiation dose imparted to the patient. Thus, AVM imparts ~3 times more cumulative frontal dose than stroke, ~4.6 times more cumulative lateral dose, and 3 times more total number of images than stroke.

**Table 1:** Dosimetric and technical data for the 3 CIs procedures, Stroke, Aneurysm and AVM.

**Parameter**	**CI**	**KAP** ^**a**^ **(Gycm**^**2**^**)**	**CDf** ^**b**^ **(mGy)**	**CDl** ^**c**^ **(mGy)**	**CD (frontal + lateral)** **mGy**	**T** ^**d**^ **(min)**	**Ni** ^**e**^	**Nr** ^**f**^
75 percentile	Stroke	133.6	562.7	227.6	797.5	23	749	23
median		78	378	118	495.7	12	442	15
min		12.1	41.5	10.6	52.1	1	20	3
max		403.3	1584	494.4	2009.3	48	2053	41
75 percentile	Aneurysm	124	881.6	282.2	1171	35	1374	26
median		85	611	95.5	801	19.5	717	19.5
min		11.3	68.3	5.7	74	2	164	4
max		625.5	4115.2	2652	6767.2	75	2588	184
75 percentile	AVM	247.6	1575.3	758.6	2384.7	48.8	1893	36
median		181	1144	537	1872	36	1375	31
min		36	123	32	204.3	4	256	7
max		417.2	4115	2652	6767.24	87	2264	59

^a^KAP: Kerma Area Product (sum of KAP of frontal and lateral C-arm units), ^b^CDf: Cumulative dose (frontal X-ray tube), ^c^CDl: Cumulative dose (lateral X-ray tube), ^d^T: Fluoroscopy time, ^e^Ni:total number of images, ^f^Nr: Total number of runs.

Based on the data above, the consortium defined DRLs as rounded values of data in Table 1.

## Discussions

In this study, we tried to develop the DRL values for the three most common INR procedures in the main public IR center in Qatar. Data show that the radiation exposure burden is lowest in S, followed by AN, and highest in AVM. As far as CI of S is concerned, treatment is done either by mechanical thrombectomy (extraction of the thrombus by a stent retriever), thrombus aspiration, or a combination (mechanical/aspiration) to regain the patency of the occluded cerebral vessel. Whatever technique may be used in the treatment, it is considered more useful with less time consumed between the start and the opening of the vessel (end of treatment) to minimize the brain ischemic injury and maintain cerebral functions. This is evidenced clearly by the short procedure times (<15 min in some patients) as well as the fluoroscopy time and radiation doses. Regarding AN, several techniques are used to treat it, such as simple coiling, stent or balloon-assisted coiling, and flow diverters, depending on the site, size, and whether the AN is ruptured or not. The procedure can be divided into two steps. First, one that includes the access, the introduction of the long sheath-guiding catheter, and the selective catheterization of the AN, which is usually located proximal along the major cerebral blood vessels. The second step is the closure of the AN by any of the above-mentioned techniques. Both steps have their difficulties and limitations and require more fluoroscopic guidance with more fluoroscopy time and radiation exposure delivered to the patient than S, especially in the second step compared to the first one. Finally, as far as AVM is concerned, treatment is more complex and technically demanding, being divided into three steps. The first is also the one that includes the access and the introduction of the base camp (long sheath-guiding catheter). The second step is the navigation of the microcatheter and microwire in the cerebral circulation to reach the malformation, which is usually located peripherally along the cerebral vessels, and the third step is the careful injection of the embolizing material to occlude the malformation completely without compromising the surrounding normal circulation. The second and third steps might extend for hours and require a lot of fluoroscopic monitoring to avoid vessel perforation and inadvertent injection of the embolizing material. Treatment of AVM is more time-consuming compared to other treatments, with a subsequent increase in fluoroscopy time and radiation exposure delivered to the patient. In the early days of the application of this treatment and with the early generation of X-ray angiography machines that had older technology and no dose reduction tools, it was not uncommon to see posttreatment transient hair loss along the portal of radiation entry that usually recovered in 2–3 months.

The study was an attempt to define DRLs based on CI rather than technique in certain cerebral INR procedures based on patient data from the single health institution in the State of Qatar where these procedures are performed. The hospital has a modern digital biplane system with experienced operators and a well-established quality control program that ensures the optimum performance of the system. These results could provide the radiation dose baseline for various cerebral CIs in the State of Qatar and for other health institutions that would like to compare their results, practices, and techniques. It is the first non-European study that defines clinical DLRs for cerebral CIs and the first one that defines these DRLs on so many technical parameters as recommended by ICRP Report 135 [12].

Comparison with other studies was extremely difficult to realize due to scarce recent literature on the subject. The EUCLID consortium identified a limited number of studies focusing on DRLs in cerebral INR procedures [16]. They concluded that comparison, even for these small numbers of studies, is quite difficult due to their inconsistency in the description of the performed procedure and the missing information of CI. Due to this lack of consistent information regarding the type of procedure, a wide range of dose and fluoroscopy time values are reported [16]. Specifically, for cerebral embolization, only a few papers were found in the recent literature reporting on DRLs based on CI rather than procedure [22, 23]. Only a broad comparison can be made which is shown in Table 2. The data of the current study are, in general, lower than those found in the recent literature. Due to the limitations identified above, it was not possible to evaluate the reasons for the differences.

**Table 2:** A comparison with the recent literature on DRLs based on CI Stroke, Aneurysm, and AVM.

**Comparison of data**		**Stroke**	**Aneurysm**	**AVM**
Qatar this study	KAP^a^ (Gycm^2^)	78	85	180
France 2017 [21]		–	190	285
Germany 2019 [22]		180	250	–
USA 2009 [23]		–	360	550
France 2011 [24]		–	349	435
Qatar this study	T^b^ (min)	12	19.5	36
France 2017 [21]		–	58	68
Germany 2019 [22]		–	–	–
USA 2009 [23]		-––	90	135
France 2011 [24]		–	58	61–
Qatar this study	Ni^c^	442	717	1375
France 2017 [22]		-––	1080	970
Germany 2019 [22]		-––	–	-––
USA 2009 [23]		-––	1350	1500
France 2011 [24]		–	1199	1410

^a^KAP, Kerma area product (sum of KAP of frontal and lateral C-arm units).

^b^T, Fluoroscopy time.

^c^Ni, Total number of images.

*–, No data.*

Some data were reported for both AN and AVM, but only DRLs for some dosimetric parameters, KAP, T, and Ni [24, 25]. Comparing our data with those literatures shows that our data is markedly low for AVM and AN (Table 2).

Hassan and Amelot [26] reported on radiation dose values for various INR procedures based on their survey and on literature data. As data are scarce for DRLs based on CI, the authors of this study attempted to perform a more general comparison with other studies that reported radiation doses in INR procedures. Due to the rapid evolution of technology (older studies have usually X-ray systems with image intensifiers, whereas more recent studies have equipment with flat panel digital detectors that impart a lower radiation dose to the patient) and dose reduction tools introduced by the industry, studies of the previous years are considered in this comparison [26–28]. The comparison again was difficult as researchers reported various dosimetric quantities using either mean or median values of KAP. This comparison is presented in Table 3 and shows that our data are again lower than those reported in the recent literature. One possible explanation could be that the X-ray system is a new technology installed in 2016 (the evolution of technology facilitates optimization of radiation dose). Another contributing factor could be that the institution has a long-established quality assurance program supported by the medical physics team that frequently also performs radiation protection training for staff that builds on a radiation safety culture within the hospital and the corporation itself.

**Table 3:** Comparisons of median radiation doses and fluoroscopy times (T) are shown for the three CIs.

	**Qatar this study**	**[26] 2017**	**[27] 2014**	**[28] 2016**	**Qatar This study**	**[26] 2017**	**[27] 2014**	**[28] 2016**
**CI**	Median KAP^a^ (Gycm^2^)				Median T^b^(min)			
**Stroke**	78	86	-––	-––	12.0	19.9	-––	–
**Aneurysm**	85	78.7	211	179	19.5	25.7	-––	44.5
**AVM**	180	149.6	338	-––	36.0	57.0	-––	–

^a^KAP, Kerma area product (sum of KAP of frontal and lateral C-arm units).

^b^T, Fluoroscopy time.

–, No data.

The clinical dose reference levels (cDRLs) for S, AN, and AVM were established specifically for HMC. These cDRLs play a crucial role in guiding clinical imaging and interventional practices at HMC and across Qatar. They serve to optimize patient dose assessment, contributing to the overall enhancement of healthcare practices in these departments.

## Conclusions

This is the first study reporting on multiple dosimetric and technical quantities for DRLs. The CIs were decided based on frequency and optimization needs in the state of Qatar. The established institutional clinical DRL values will further facilitate patient dose optimization and quality improvement processes within HMC. To ensure that patient doses are as low as reasonably achievable, specific protocols tailored to patient clinical indications should be maintained regularly.
